# Effect of dosimeter’s position on occupational radiation extremity dose measurement for nuclear medicine workers during ^18^F-FDG preparation for PET/CT

**DOI:** 10.1186/s40658-016-0152-5

**Published:** 2016-08-05

**Authors:** Fabien Salesses, Paul Perez, Aline E. Maillard, Julie Blanchard, Sabine Mallard, Laurence Bordenave

**Affiliations:** 1 Médecine Nucléaire, CHU de Bordeaux, F-33000 Bordeaux, France; 2 USMR, CHU de Bordeaux, F-33000 Bordeaux, France; 3 Pôle d’Imagerie Médicale, CHU de Bordeaux, F-33000 Bordeaux, France; 4 Bioingénierie Tissulaire, U1026, Univ. Bordeaux, F-33000 Bordeaux, France

**Keywords:** Radiation dosimetry, Extremity, ^18^F-FDG, Operational dosimeter

## Abstract

**Background:**

The recent spread of positron emission tomography-computed tomography (PET/CT) poses extremity dosimetry challenges. The question arose whether the radiation dose measured by the ring thermoluminescent dosimeter usually worn on the proximal phalanx (P1) of the index finger measures doses that are representative of the true doses received by the upper extremities of the operators. A prospective individual dosimetry study was performed in which the personal equivalent dose Hp (0.07) received during a specific 2-[^18^F]fluoro-2-deoxy-d-glucose (^18^F-FDG) manual dose-dispensing procedure was measured in a paired design by two operational personal electronic dosimeters fitted on the palm side of the index finger, namely in the P1 and distal phalanx (P3) positions. The study participants were ten nuclear medicine technologists working in two nuclear medicine departments. The personal equivalent radiation doses received by the palm side of the proximal phalanx of the index finger [Hp (0.07)_P1_] and that received by the distal phalanx [Hp (0.07)_P3_] were compared.

**Results:**

The median Hp (0.07)_P3_/Hp (0.07)_P1_ ratio per participant varied between 1.0 and 2.5 (based on 23 to 31 measurements per participant). The 271 paired measurements revealed a crude Hp (0.07)_P3_/Hp (0.07)_P1_ ratio of 1.67, significantly different from 1 (*p* = 0.0004, 95 % CI [1.35–2.07]). When adjusted on participant’s gender and mother vial activity, the ratio was similar (1.53, *p* = 0.003, 95 % CI [1.22–1.92]).

**Conclusions:**

The study demonstrated a significant disparity that may exist between the radiation doses measured in the P1 and P3 positions of operators during ^18^F-FDG manipulation. These findings emphasize the importance of performing workplace dosimetry studies adapted to each radiopharmaceutical and manipulation thereof, aiming to guarantee optimal workers’ dosimetry monitoring schemes.

**Trial registration:**

Hospital Nursing and Paramedical Research Program (PHRIP, 2011–2013) from the French Ministry of Health (DGOS), http://social-sante.gouv.fr/IMG/pdf/Resultats_PHRIP_2011.pdf

## Background

The spread of positron emission tomography-computed tomography (PET/CT) and the expansion of its indications imply the preparation of an ever-growing number of positron-emitting radiopharmaceuticals. 2-[^18^F]Fluoro-2-deoxy-d-glucose (^18^F-FDG) is by far the most widely used radionuclide in PET imaging. It induces exposure to high-energy ionizing radiation, hence challenges in radiological protection of medical staff.

According to the Council Directive Euratom of the EU [1], the ICRP, and the ICRU [2, 3], transposed into the French Labor Code [4], the limit of radiation (effective) dose to the whole body is 20 mSv over 12 consecutive months. If the dose to any part of the extremities of a worker is likely to exceed three tenths of the annual dose limit, an additional dosimeter should be placed on the part of the extremity where the dose is expected to have its highest value. In practice, extremity monitoring is carried out by measuring the personal dose equivalent Hp (0.07) [3], the annual limit of which is 500 mSv.

The present study is the first one sponsored by the French Ministry of Health (DGOS) carried out by a team of nuclear medicine (NM) technologists (NMTs); it was performed in the framework of the French Hospital Nursing and Paramedical Research Program (PHRIP 2011) with the objective of ensuring appropriate workers’ safety in NM departments with adherence to the ALARA safety principle.

The pertinent French ministerial order [5] recommends setting up the means of adapted individual dosimetry but leaves open to interpretation of the modalities of wearing extremity dosimeters, which has sprouted a variety of routine practices and discrepant workplace dosimetry studies. The thermoluminescent dosimeters (TLDs) routinely used are not ideally adapted to NM in its entirety, namely, the ring TLD measures the radiation always in the same position and reflects the average dose received monthly from a multitude of manipulations of radiopharmaceuticals. Consequently, there is uncertainty whether the radiation measured by the passive TLDs worn on the first phalanx of the index finger represents the true extremity exposure in NMTs who manipulate ^18^F-FDG, hence the rationale for a study designed to determine which anatomical location of the index finger would receive the higher radiation dose. Up to now, no published large-scale study has employed electronic personal dosimeters (EDs), allowing for real-time dose measurements with a much lower detection threshold and a better sensitivity than TLDs.

The primary objective of the study was to prospectively estimate the difference between the radiation doses, measured by two operational EDs, received by the palm side of the proximal (first) phalanx (P1) of the index finger [Hp (0.07)_P1_] and that received by the distal (third) phalanx (P3) of the same finger [Hp (0.07)_P3_] during a manual drawing into a syringe of a ^18^F-FDG dose. The secondary objectives consisted of studying the impact of radiopharmaceutical characteristics and demographic data on the Hp (0.07)_P3_/Hp (0.07)_P1_ ratio.

## Methods

### Study design

This prospective dosimetry study focused on measuring the personal equivalent dose Hp (0.07) received during a specific ^18^F-FDG dose drawing procedure. This was performed in a paired design with two operational EDs fitted on the palm side of the index finger, namely in the P1 and P3 positions.

The study was approved by several committees in 2012, i.e., the Institutional Review Board (CPP Sud-Ouest Outre-Mer), the French Consultative Committee for Data Processing in Health Research (CCTIRS), and the French National Commission on Information Technology and Civil Liberties (CNIL).

### Participants

The study participants were ten NMTs, all right-handed, who have worked regularly in ^18^F-FDG preparation in the NM departments of the Bordeaux University Hospital and the Côte Basque-Bayonne Hospital, each employing five study subjects. Taking into account that whatever the used hand (dominant or non-dominant), the gesture is realized identically, because of the hot cell layout and the kind of device used. All participants signed an informed consent before enrollment. Demographic data were collected from each participant.

### Dosimetry procedures

We used Unfors® NED electronic operational dosimeters (Unfors Instruments AB, Billdal, Sweden) equipped with semiconductor sensor technology, whose technical measuring characteristics are fully adapted to *γ* radiation: dose rate range 0.18 mSv/h–9.0 Sv/h, dose range 50 nSv–9999 Sv, start trigger level 0.27 mSv/h, and end trigger level 0.18 mSv/h. The EDs underwent mandatory annual calibration by the manufacturer. The uncertainty measure of the ED stipulated on the calibration certificate issued by the manufacturer was 6 %.

The advantages of the EDs consisted of a direct reading of the measured dose and lack of reading delay typical of TLDs, along with the latter’s risk of loss, damage, and negative results displayed when below detection threshold (i.e., 100 μSv with ring TLDs).

**Fig. 1:**
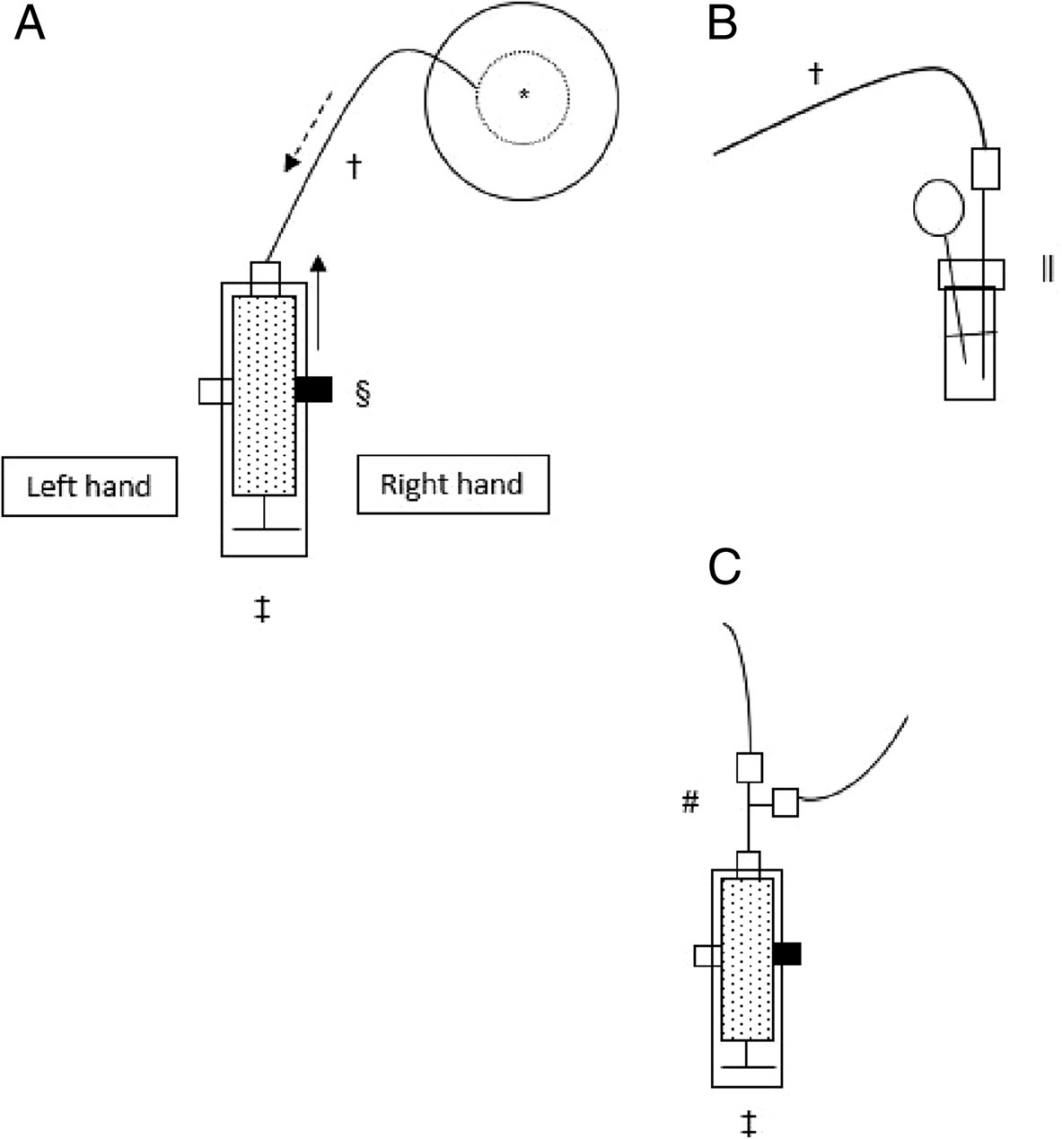
The manual drawing of the ^18^F-FDG dose into the syringe. **a** Whole device inside the hot cell: *Activimeter (multidose ^18^F-FDG vial inside); †30-cm extension tube; ‡syringe inside tungsten shield syringe; §thumbscrew holding the syringe firmly in place inside the tungsten shield; *black arrow* refers to the direction of thumbscrew manipulation for drawing. *Dotted arrow* refers to the ^18^F-FDG aspiration from the vial inserted in the activimeter. Hands are placed. **b** || Multidose to the ^18^F-FDG vial (inside the activimeter). **c** #Three-way stopcock connected to the syringe inside the tungsten shield

The radiation measurement focused on the process of ^18^F-FDG dispensing by manual drawing into a syringe (Fig. 1).

**Fig. 2:**
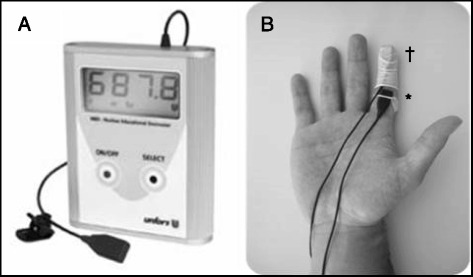
**a** The Unfors® NED operational dosimeter. **b** Positions of the dosimeter sensors: *P1 (proximal); †P3 (distal)

The radiation measuring process comprised the following working steps: the sensors were fitted onto the palm side of the operator’s P1 and P3 (Fig. 2); the two dosimeters were turned on; the operator’s hands, protected by disposable non-sterile gloves, were placed into the glove port of the high-energy hot cell (Lemer Pax in Bayonne; Medisystem in Bordeaux); the tungsten shield syringe complex was fitted onto the 30-cm extension tube (the opposite end of which had been previously connected to the needle inserted into the multidose vial into which a vent needle fitted to a vent filter had also been inserted prior to the radiation measurement phase); the dose was dispensed by manual drawing into the syringe; the excess air was purged; the syringe was disconnected from the extension tube; the three-way stopcock-Y-connector combo was fitted onto the syringe; the operator’s hands were withdrawn from the glove port; and the two dosimeters were turned off and the study dosimetry ended. The choice fell on this truncated manipulation sequence for reasons of maximal reproducibility between the two investigational centers. While the utilized material and the modus operandi were identical in the two departments, there were minor layout differences, namely in the transfer of ^18^F-FDG out of the hot cell, hence the decision to stop the study measurement after the fitting the three-way stopcock.

Into the case report form, we transcribed the dose displayed by the EDs and additional parameters of interest for their potential impact on the disparity between Hp (0.07)_P3_ and Hp (0.07)_P1_: volume and activity of the multidose ^18^F-FDG vial, volume and activity of the withdrawn ^18^F-FDG dose, degree of discomfort caused by wearing the two sensors, the hand used for connecting the three-way stopcock to the syringe, and the duration exposure of each detector.

### Statistical analysis

The study was powered to detect a minimum difference of 35 μSv between Hp (0.07)_P1_ and Hp (0.07)_P3_, with a variance of 120 μSv, according to a pilot study realized with only one ED and a NMT (unpublished data), a two-tailed type I error (*α*) of 0.05 and a power (1-*β*) of 90 %; the conservative hypothesis of no correlation between the doses received by the two phalanges was assumed. The calculation resulted in a requirement of at least 126 paired measurements (nQuery Advisor v6.0; Statistical Solutions Ltd., Cork, Ireland), but this number was brought to 200, in order to allow analysis of the explanatory variables impacting the disparity in radiation incurred by the two phalanges.

The disparities between Hp (0.07)_P3_ and Hp (0.07)_P1_ were estimated by a mixed-effects linear regression model. Logarithmic transformation of the measured dose was required in order to respect the model’s assumption of normal distribution of residuals. The factors associated with the disparity between the doses received by the two phalanges were selected by univariable analysis (at *p* value of <0.2) and were entered into the multivariable mixed model. All non-significant factors were eliminated by backward stepwise selection (at *p* value of <0.05) and verification that those factors were not confounders. The regression model is described by the following equation:

$$ \log \left(\mathrm{H}\mathrm{p}\ {(0.07)}_{\mathrm{P}3}\right)- \log \left(\mathrm{H}\mathrm{p}\ {(0.07)}_{\mathrm{P}1}\right)=\mathrm{intercept}+{\beta}_1\times {\mathrm{factor}}_1+\dots +{\beta}_i\times {\mathrm{factor}}_i $$

However, it is more straightforward when it is rewritten as

$$ \frac{\mathrm{Hp}\ {(0.07)}_{\mathrm{P}3}}{\mathrm{Hp}\ {(0.07)}_{\mathrm{P}1}}={e}^{\mathrm{intercept}}\times {e}^{\beta_1\times {\mathrm{factor}}_1}\times \dots \times {e}^{\beta_i\times {\mathrm{factor}}_i} $$

Thus, the exponentiated regression coefficients (*β*_*1*_,…, *β*_*i*_) estimate the per unit increases in the value of the Hp (0.07)_P3_/Hp (0.07)_P1_ ratio.

All statistical analyses were performed with SAS software, version 9.3 (SAS Institute, Cary, NC, USA).

We had performed several preliminary studies that verified a good ED reliability.

Operators routinely perform the manipulation sequence almost identically regardless of their handedness, except in the last step of the study sequence when the right and left hands were interchangeably used to fit the three-way stopcock–Y-connector combo onto the syringe. Therefore, the hand used in that last step was included as a potential explanatory variable in the mixed-effects linear model.

## Results

The study was carried out between September 2012 and February 2013 with ten participating NMTs (eight females and two males) with a mean age (standard deviation (SD)) of 33.1 [±9.7] years, all right-handed. Their mean (±SD) seniority in the NMT profession and at the PET/CT station was 130.1 [±125.5] months and 61.9 [±23.2] months, respectively. A total of 287 paired measurements were carried out. Twenty measurements were fraught with incidents and 16 of those were excluded from the final dataset (15 at Bordeaux and 1 at Bayonne). The incidents leading to the exclusion from the analysis had been caused by either human errors consisting prematurely stopped measurements or were technical in nature, such as dosimeter breakdown or failure necessitating repair or replacement. Thus, the final analysis dataset comprised 271 valid measurements (137 at Bordeaux and 134 at Bayonne).

The results of all variables pertaining to the ^18^F-FDG drawing sequence are presented in Table 1.

**Table 1:** Variables related to the radioactivity of the multidose vial, withdrawn ^18^F-FDG dose, and the manual drawing maneuver

Variable	*N* (%)	Mean (SD)	Min; max
Activity of ^18^F-FDG multidose vial (MBq)	–	985.1 (519.7)	200.0; 3774.0
Volume of ^18^F-FDG in multidose vial (mL)	–	5.7 (1.9)	1.9; 10.4
Specific activity of multidose vial (MBq/mL)	–	171.6 (73.1)	34.1; 553.7
Activity of withdrawn ^18^F-FDG dose (MBq)	–	295.1 (63.4)	156.0; 554.0
Volume of withdrawn ^18^F-FDG dose (mL)	–	2.0 (0.7)	0.3; 4.1
Discomfort in flexing the index finger caused by sensors			
None	3 (30)	–	
Mild	5 (50)		
Severe	2 (20)		
Hand fitting the 3-way stopcock onto the syringe			
Right	8 (80)	–	–
Left	2 (20)		
Exposure duration (s)			
First phalanx (P1) sensor	–	51.5 (30.5)	19.1; 343.4
Third phalanx (P3) sensor		51.8 (30.3)	15.8; 344.4

*SD* standard deviation

**Fig. 3:**
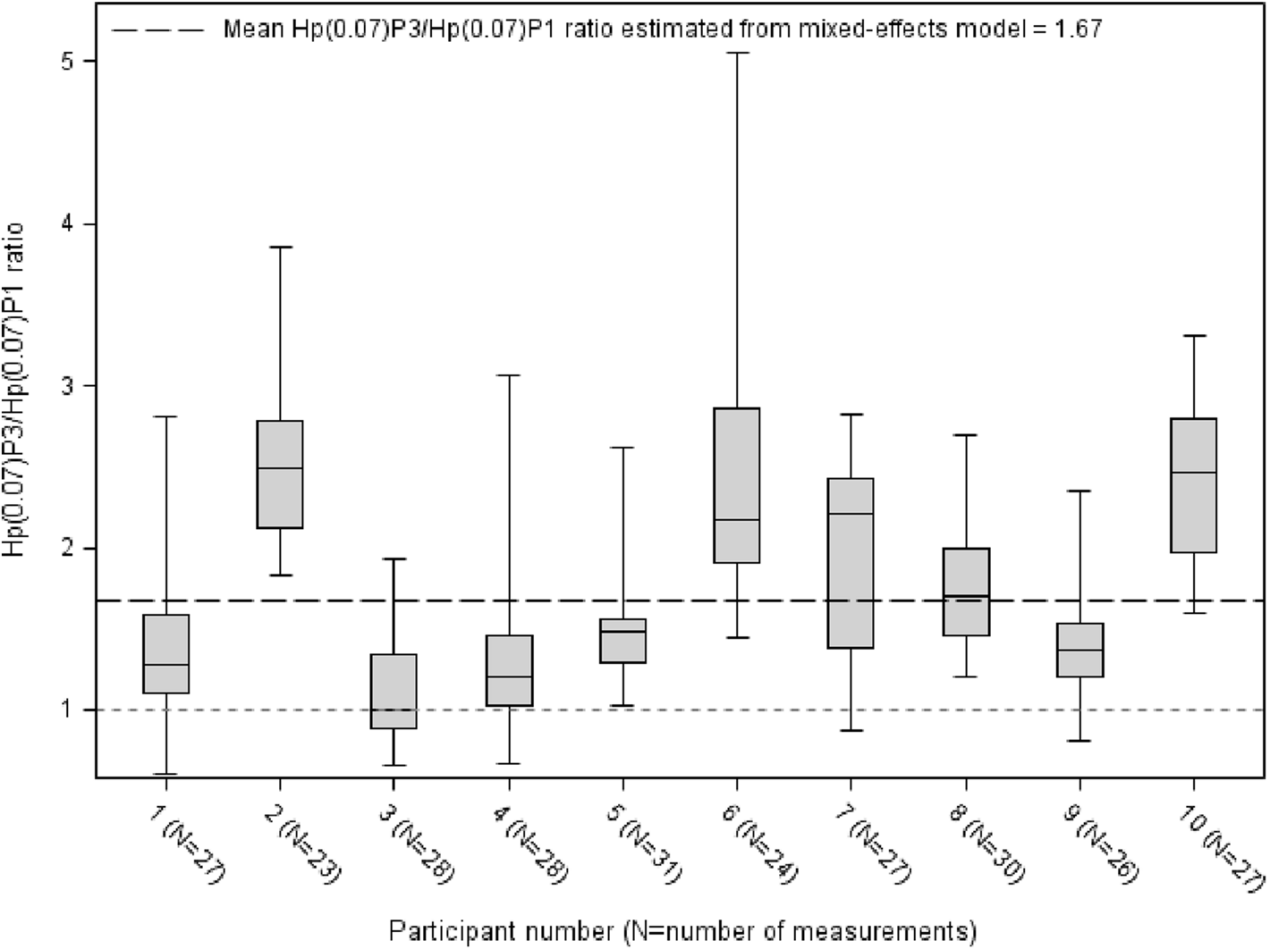
Interquartile range box plot of the distribution of the Hp (0.07)_P3_/Hp (0.07)_P1_ ratio (between the radiation dose received by the palm side of the distal phalanx and the proximal phalanx of the index finger) per participant. On the ordinate: the Hp (0.07)_P3_/Hp (0.07)_P1_ ratio in *N* = 271 paired observations performed in ten study participants. On the *abscissa*: the nominal number of the study participant (the number of repeated measurements per participant)

The geometric mean crude Hp (0.07)_P3_/Hp (0.07)_P1_ ratio (calculated as if the 271 measures had been independent) was 1.65 (Table 2), which was very close to that yielded by the mixed-effects linear regression model (that accounted for intra-participant correlation), i.e., 1.67, significantly different from 1 (*p* = 0.0004, 95 % CI [1.35–2.07]). Based on 23 of 31 measurements per participant, the median Hp (0.07)_P3_/Hp (0.07)_P1_ ratio per participant varied between 1.0 and 2.5 (Fig. 3). Four participants had median Hp (0.07)_P3_/Hp (0.07)_P1_ ratios higher than 2. This means that at least 100 % more radiation dose was absorbed by P3 than P1 in those patients. The range in radiation doses Hp (0.07) measurements was 6.9 to 321.4 μSv for Hp (0.07)_P1_ and 6.5 to 681.6 μSv for Hp (0.07)_P3_ (Table 2).

**Table 2:** Description of the Hp (0.07)_P1_ and Hp (0.07)_P3_ radiation doses received by the proximal and distal phalanges of the index fingers, respectively, in ten study participants, based on 271 paired observations; the ratios and the differences between these two parameters

	Radiation doses Hp (0.07) (μSv)		Hp (0.07)_P3_/Hp (0.07)_P1_ ratio	Hp (0.07)_P3_–Hp (0.07)_P1_ difference (μSv)
	Hp (0.07)_P1_	Hp (0.07)_P3_		
Arithmetic mean (SD)	46.67 (42.6)	81.01 (85.4)	1.77 (0.7)	34.34 (52.0)
Geometric mean (coefficient of variation, %)	35.49 (0.8)	58.46 (0.9)	1.65 (0.4)	NA
Median (first; third quartiles)	34.25 (22.9; 54.1)	60.03 (34.3; 92.7)	1.58 (1.3; 2.2)	19.55 (8.4; 39.0)
Minimum; maximum	6.9; 321.4	6.5; 681.6	0.6; 5.1	−29.3; 368.0

*SD* standard deviation, *NA* not applicable (negative values)

The variables that showed a statistically significant influence on the Hp (0.07)_P3_/Hp (0.07)_P1_ ratio in multivariable analysis were as follows: (1) the radiation activity of the mother vial, *e*^*β*^ = 1.01, *p* = 0.0002, signifying a slight practical increase in Hp (0.07)_P3_/Hp (0.07)_P1_ by a multiplicative factor of 1.01 for every 10 MBq/mL increase in the radiation activity of the mother vial and (2) sex, *e*^*β*^ = 1.59, *p* = 0.036, meaning that the value of Hp (0.07)_P3_/Hp (0.07)_P1_ in males was 59 % higher than in females. When adjusted to these two factors, the Hp (0.07)_P3_/Hp (0.07)_P1_ ratio marked 1.53 (*p* = 0.003, 95 % CI [1.22–1.92]) (Table 3).

**Table 3:** Results of univariable and multivariable mixed-effects linear regression of the Hp (0.07)_P3_/Hp (0.07)_P1_ ratio of doses received by the distal (P3) and proximal (P1) phalanges of the index finger, based on 271 paired observations in ten study participants

Variable^a^	Univariable analysis		Multivariable analysis	
	*e* ^*β*^ [95 % CI]	*p* value	*e* ^*β*^ [95 % CI]	*p* value
Intercept^b^			1.53 [1.22–1.92]	0.003
Hospital center				
Bordeaux Hospital	0.83 [0.57–1.21]	0.342		
Bayonne Hospital (ref)	–			
Specific activity of multidose vial (10 MBq/mL)	1.01 [1.01–1.02]	<0.001	1.01 [1.00–1.10]	<0.001
Specific activity of drawn dose (10 MBq/mL)	1.01 [1.00–1.01]	<0.001		
Age (years)	0.99 [0.97–1.02]	0.606		
Gender				
Male	1.54 [1.04–2.28]	0.030	1.59 [1.03–2.46]	0.036
Female (ref)	–			
Seniority of NMT in profession (months)	0.99 [0.99–1.00]	0.596		
Seniority of NMT in PET/CT station (months)	1.00 [0.99–1.01]	0.601		
Hand fitting the 3-way stopcock onto the syringe				
Right	0.81 [0.51–1.30]	0.376		
Left (ref)	–			
Discomfort in flexing the index finger				
None to mild	1.01 [0.62–1.66]	0.973		
Severe (ref.)	–			
Discomfort in flexing the index finger				
None	1.41 [0.98–2.01]	0.062		
Mild to severe (ref.)	–			

^a^For continuous variables, the exponential function of the regression coefficient *e* ^*β*^ is the multiplicative factor by which the value of Hp (0.07)_P3_/Hp (0.07)_P1_ is changed with a one-unit increase in the value of that variable. For two-level categorical variables, one level versus the other level multiplies the value of Hp (0.07)_P3_/Hp (0.07)_P1_ by *e* ^*β*^

^b^The intercept (or constant) is the mean value of Hp (0.07)_P3_/Hp (0.07)_P1_ for a mean specific activity of the multidose vial in females (the reference for gender)

## Discussion

The aim of this prospective study was to perform a workplace dosimetry study only focused on ^18^F-FDG preparation, in view of defining the most relevant position of dosimeter for extremity dose measurement in the targetted manipulation. We provide a knowledge gain to the community by describing extensively the specific preparation procedure and using sensitive EDs.

This study showed that the radiation dose received by the distal phalanx of the index finger during a definite manual drawing sequence of ^18^F-FDG doses destined for PET imaging was significantly higher by 67 % on average than the one received by the proximal phalanx. This result may portend, in the clinical routine, an important potential difference between the data of standard radionuclide exposure monitoring based on classic individual dosimetry in the proximal phalanx and the estimated radiation received by the distal phalanx. As expected, the position of the extremity dosimeter has a crucial impact in extremity radiation monitoring in NMTs who perform manual dispensing of ^18^F-FDG doses for PET.

A large range in Hp (0.07) measurements (for P1 and P3) was obvious (Table 2). Explanations for that could be that (i) the manual dispension is not totally reproducible for a given participant and between participants in contrast to semi-automatic and automatic dispension and (ii) the specific activity is greatly variable from one dispension to another.

The specific activity (MBq/mL) of the multidose vial had a statistically significant impact, albeit limited in the clinical routine, on the Hp (0.07)_P3_*vs.* Hp (0.07)_P1_ disparity, which could be explained by an increased difficulty of manually drawing a set value of radioactivity (MBq) in smaller volumes (mL). The influence of the operator’s gender is more challenging to interpret given the 8:2 male-to-female ratio and the small size of the population, yet, the primary objective was the analysis of the dose disparity between the two monitoring positions, not the population-related variables.

Despite their user-friendliness, operational EDs entail some unwieldiness that may not strictly emulate the routine practice conditions. This discomfort might have altered the routine radionuclide dispensing sequence to some extent and influenced the local skin dose disparity between the two finger positions. Nonetheless, the subjective sensor-related discomfort had no statistically significant impact on dose disparity between the finger positions. The exposure duration was similar in the P1 and P3 sensor positions; consequently, it did not generate supplementary irradiation. This study revealed some wearing difficulty of the two operational ED sensors, hence the necessity to accurately define the ideal position of the sensor when a unique dosimeter is used in workplace dosimetry studies that attempt to measure the maximum local extremity dose.

No extrapolation of our results to yearly radiation doses was feasible because of the specific dispensing sequence used in the study, which amounted to a mere fraction of the total dose received by the extremities during general work with radiopharmaceuticals.

There was an important intra-participant disparity in the Hp (0.07)_P3_/Hp (0.07)_P1_ ratio: its median intra-participant value varied between 1.0 and 2.5, demonstrating dose differences of more than 100 % in four of the ten study participants. If a 67 % discrepancy existed between the dose received by the position routinely used in dosimetry monitoring [Hp (0.07)_P1_)] and a higher dose measured in this study in the distal position [(Hp (0.07)_P3_)] in every radiopharmaceutical preparation or administration field, there would be a risk of attaining or even exceeding the annual radiation exposure limit of 500 mSv for extremities, leaving little margin of safety for potential exposure accidents. This finding carries all the more significance with the high-dose differences between P3 and P1 (>100 %) that were found in four of ten study participants (Fig. 3) and emphasizes the importance of dosimetry monitoring. The underestimated doses by a factor of at least 2 justify routine overestimations destined to counterbalance the underestimation of the skin doses measured under routine conditions in the first phalanx position. These findings are correlated with the recommendation of the European collaborative project Optimization of Radiation Protection for Medical Staff (ORAMED) to multiply by a factor of 6 the reading of the TLD when placed on the palm side of the P1 position of the non-dominant hand in order to estimate the maximum local skin dose [6].

The strength of the study resided in (1) the paired design of dose measurements performed during the same sequence of manual ^18^F-FDG drawing (which proved a crucial element in light of the heterogeneity of the absolute and specific radioactivity parameters between different repeated measurements (Table 1)); (2) the use of operational EDs with higher sensitivity than that of passive TLDs; (3) the real-time reading of radiation doses; and (4) the highest total of paired measurements (271) ever published in the hitherto available literature on this topic.

The limitation of the study consisted of the discomfort caused by wearing two sensors on the index finger, although traded off against the valuable paired measurement design and the lack of prolongation of the habitual ^18^F-FDG manipulation sequence. The low number of study participants (ten operators) limited the analysis of human explanatory variables (e.g., the influence of gender).

The most recent report of the French Nuclear Safety Authority in 2014, which had evaluated the activity of NM departments in France between 2009 and 2011, concluded that despite the preponderant radiation exposure of extremities, notably in PET/CT work, current extremity dosimetry practices lack in uniformity and their results are seldom exploited [7]. Moreover, the conclusions of ORAMED, which aimed to improve extremity dosimetry in nuclear medicine with special emphasis on PET, recommend placing the ring dosimeter with its sensitive part oriented towards the palm side of the index finger’s first phalanx (P1) of the non-dominant hand [6]. The results obtained in the present well-defined dispensing sequence of ^18^F-FDG demonstrated that such a general recommendation should be liable to variations. The variety of manual dispensing techniques specific to each radiopharmaceutical presumably implies a variable anatomic location of the maximum local skin dose. Thus, the design of the present study revealed the need to perform rigorous workplace extremity dosimetry studies with materials (e.g., operational EDs in our study) but mainly position choices (of the hand, finger, and phalanx) that are best suited for the particular dispensing maneuver of interest. In the particular case of ^18^F-FDG preparation, positioning the sensor on the palm aspect of the third phalanx of the right index finger would be most sensible.

By virtue of their experience and sharpened critical eye on their own practices, NMTs that perform a dosimetry study focusing on a specific manipulation should be consulted in order to target the potential location of maximum local skin dose. This should be always done in conjunction with findings of prior studies performed either on the premises or published in the literature.

The most pertinent means of extremity monitoring dosimetry should be implemented in every department in the interest of the staff working in the preparation and administration of radioactive sources. The everyday approach should always tend to reduce radiation exposure, as opposed to the mere contentment with remaining within admissible radiation exposure limits, since the latter have been constantly revised downwards. Protection devices may progress considerably and allow further radiation exposure reduction, notably of the extremities. In this regard, the use of semi-automated syringe drawing devices and injectors in everyday practice is fully justified. Simultaneously wearing ring TLDs on both dominant and non-dominant hands could improve the evaluation of the true maximal dose received globally by the upper extremities of workers, without additional requirements. It is imperative to maximally reduce the received doses in order to attenuate the gap between the TLD-measured average radiation doses and those that are actually received by the skin *via* regular revision and analysis of existent practices and optimal equipment in workplaces (materials, ergonomics, screens, etc). At the organizational level, a judicious staff turnover at workstations is essential because of the ever-growing activity of manipulating positron-emitting radiopharmaceuticals.

Our conclusions complement those of authoritative reference studies in the field of radiation protection dosimetry [6–13] and underscore the emphasis laid on extremity radiation monitoring [6–9, 11–13].

## Conclusions

Prevention constitutes a major axis in the protection of radiopharmaceutical-manipulating personnel. The present study in occupational radiation protection demonstrated a significant potential disparity (67 %) between radiation doses measured in the proximal and distal phalanx positions of the index finger (upper in the distal position P3). Our findings emphasize the importance of the choice of the extremity dosimeter sensor’s position during radiation monitoring (i.e., the hand, finger, phalanx) and the requirement to perform workplace dosimetry studies adapted to each radiopharmaceutical and manipulation. Such workplace dosimetry studies influence the implementation of devices and NMT staff. These studies need to be conducted jointly by all actors involved in implementing radiation protection and workers’ safety, i.e., not only radiation safety officers, but NMTs as well, the latter exerting a critical and analytical view, as required by the good health-care practices in NM.
